# (*R*,*R*)-(*N*,*N*′-Diferrocenylcyclo­hexane-1,2-diyldiimino)dibenzonitrile

**DOI:** 10.1107/S1600536809035788

**Published:** 2009-09-12

**Authors:** Fang Chen

**Affiliations:** a Ordered Matter Science Research Center, College of Chemistry and Chemical Engineering, Southeast University, Nanjing 210096, People’s Republic of China

## Abstract

In the title compound, [Fe_2_(C_5_H_5_)_2_(C_34_H_34_N_4_)], two ferrocenes are bridged by a cyclo­hexane-1,2-diamine unit. The cyclo­penta­dienyl rings of the two ferrocene units are almost parallel [dihedral angles of 0.7 (4) and 1.0 (4)° in the two units] and eclipsed, as is typically found for similar monosubstituted ferrocene derivatives. The dihedral angle formed by the two benzene rings is 20.2 (3)°. The Fe—C bond lengths to the two substituted Cp rings vary from 2.014 (4) to 2.070 (3) Å, and are in the normal range. In the crystal, mol­ecules are linked by C—H⋯N inter­actions, forming an infinite two-dimensional network.

## Related literature

For the applications of ferrocene derivatives, see: Yang *et al.* (2002 ▶); Roberto *et al.* (2000 ▶); Long (1995 ▶). For related structures, see: Hess *et al.* (1999 ▶); Base *et al.* (2002 ▶); For the synthetic strategy, see: Cho *et al.* (1999 ▶); Sutcliffe *et al.* (2002 ▶).
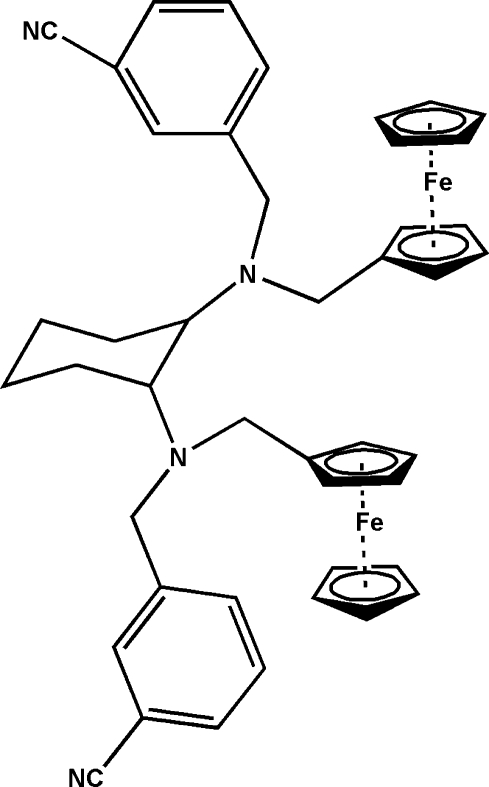


## Experimental

### 

#### Crystal data

[Fe_2_(C_5_H_5_)_2_(C_34_H_34_N_4_)]
*M* *_r_* = 740.53
Monoclinic, 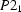

*a* = 10.302 (2) Å
*b* = 17.573 (4) Å
*c* = 10.746 (2) Å
β = 108.40 (3)°
*V* = 1846.0 (7) Å^3^
*Z* = 2
Mo *K*α radiation
μ = 0.82 mm^−1^
*T* = 298 K
0.50 × 0.40 × 0.40 mm

#### Data collection

Rigaku SCXmini diffractometer
Absorption correction: multi-scan (*CrystalClear*; Rigaku, 2005 ▶) *T*_min_ = 0.606, *T*_max_ = 0.720
19480 measured reflections
8770 independent reflections
6719 reflections with *I* > 2σ(*I*)
*R*_int_ = 0.039

#### Refinement

*R*[*F* ^2^ > 2σ(*F* ^2^)] = 0.063
*wR*(*F* ^2^) = 0.171
*S* = 1.08
8770 reflections
391 parameters
1 restraint
All H-atom parameters refined
Δρ_max_ = 0.64 e Å^−3^
Δρ_min_ = −0.39 e Å^−3^
Absolute structure: Flack (1983 ▶), 4198 Friedel pairs
Flack parameter: 0.004 (21)

### 

Data collection: *CrystalClear* (Rigaku, 2005 ▶); cell refinement: *CrystalClear*; data reduction: *CrystalClear*; program(s) used to solve structure: *SHELXS97* (Sheldrick, 2008 ▶); program(s) used to refine structure: *SHELXL97* (Sheldrick, 2008 ▶); molecular graphics: *SHELXTL* (Sheldrick, 2008 ▶); software used to prepare material for publication: *SHELXTL*.

## Supplementary Material

Crystal structure: contains datablocks I, global. DOI: 10.1107/S1600536809035788/su2133sup1.cif

Structure factors: contains datablocks I. DOI: 10.1107/S1600536809035788/su2133Isup2.hkl

Additional supplementary materials:  crystallographic information; 3D view; checkCIF report

## Figures and Tables

**Table 1:** Hydrogen-bond geometry (Å, °)

*D*—H⋯*A*	*D*—H	H⋯*A*	*D*⋯*A*	*D*—H⋯*A*
C21—H21*A*⋯N1^i^	0.97	2.59	3.486 (9)	154
C37—H37⋯N2^ii^	0.93	2.59	3.364 (11)	141

Symmetry codes: (i) 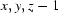; (ii) 
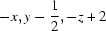
.

**Fig. 1.**
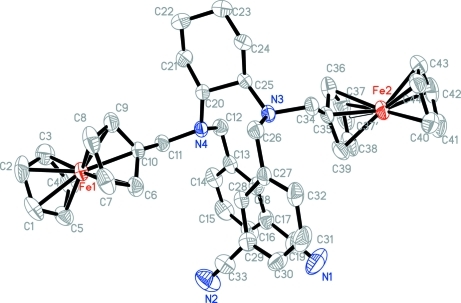
A view of the molecular structure of the title compound, with displacement ellipsoids drawn at the 30% probability level [H-atoms have been omitted for clarity].

## supplementary crystallographic information

### Comment

The chemistry of ferrocene has received much attention because of its applications in many fields, such as in catalysis (Yang *et al.*, 2002), organic or organometallic synthesis and materials, non-linear optical (NLO) materials (Long, 1995; Roberto *et al.*, 2000), and medicinal materials. As a part of our ongoing investigations of ferrocene derivatives we have prepared the title compound and report herein on its structure in the solid state.

The molecular structure of the title compound is illustrated in Fig. 1, and the geometrical parameters are given in the archived CIF. The cyclopentadienyl rings in the two ferrocene moieties (involving atoms Fe1 and Fe2) are almost parallel, with dihedral angles of 0.7 (4) ° and 1.0 (4) °, respectively, and eclipsed as viewed down the normal to the Cp ring. The cyclohexane ring has a chair configuration and the two ferrocenemethylamino groups are equatorially bonded to it, as expected. The dihedral angle formed by the two benzene rings is 20.2 (3)°. The Fe—C bond lengths to the two substituted Cp ring vary from 2.014 (4) to 2.070 (3) Å, and are in agreement with the values reported for related compounds (Hess *et al.*, 1999; Base *et al.*, 2002).

In the crystal the molecules are linked by C-H···N interactions, involving the nitrile N-atoms, so forming an infinite two-dimensional network (Table 1).

### Experimental

The preparation of the precursors of the title compound has been reported on previously (Cho *et al.*, 1999; Sutcliffe *et al.*, 2002). For the preparation of the title compound, K_2_CO_3_ (10 mmol) was added to a mixture of (*R*,*R*)-*N*,*N*'-Bis(ferrocenylmethyl)cyclohexane-1, 2-diamine (5 mmol) and 3-(bromomethyl)benzonitrile (10 mmol) in acetone (40 ml), and the mixture was heated to reflux for 6 h. The mixture was then cooled, filtered, and the filtrate evaporated to dryness. The yellow solid obtained was recrystallized from a mixture of acetone and petroleum ether (yield: 68%). Yellow crystals, suitable for X-ray diffraction analysis, were obtained by slow evaporation of a dichloromethane solution at rt after 3 days.

### Refinement

The H-atoms were included in calculated positions and treated as riding atoms: C—H = 0.93 - 0.98 Å, with U_iso_(H) = 1.2U_eq_(parent C-atom). The EADP's of certain atoms were made equal during the refinement [EADP C1 C2 C3 C4 C5; EADP C36 C37 C38 C39 C35; EADP C22 C23 C21].

### Crystal data

**Table:**

[Fe_2_(C_5_H_5_)_2_(C_34_H_34_N_4_)]	*F*(000) = 776
*M**_r_* = 740.53	*D*_x_ = 1.332 Mg m^−^^3^
Monoclinic, *P*2_1_	Mo *K*α radiation, λ = 0.71073 Å
Hall symbol: P 2yb	Cell parameters from 4276 reflections
*a* = 10.302 (2) Å	θ = 3.0–27.5°
*b* = 17.573 (4) Å	µ = 0.82 mm^−^^1^
*c* = 10.746 (2) Å	*T* = 298 K
β = 108.40 (3)°	Prism, yellow
*V* = 1846.0 (7) Å^3^	0.50 × 0.40 × 0.40 mm
*Z* = 2	

### Data collection

**Table:**

Rigaku SCXmini diffractometer	8770 independent reflections
Radiation source: fine-focus sealed tube	6719 reflections with *I* > 2σ(*I*)
graphite	*R*_int_ = 0.039
Detector resolution: 13.6612 pixels mm^-1^	θ_max_ = 28.0°, θ_min_ = 2.3°
ω scans	*h* = −13→13
Absorption correction: multi-scan (*CrystalClear*; Rigaku, 2005)	*k* = −22→23
*T*_min_ = 0.606, *T*_max_ = 0.720	*l* = −14→14
19480 measured reflections	

### Refinement

**Table:**

Refinement on *F*^2^	Secondary atom site location: difference Fourier map
Least-squares matrix: full	Hydrogen site location: inferred from neighbouring sites
*R*[*F*^2^ > 2σ(*F*^2^)] = 0.063	All H-atom parameters refined
*wR*(*F*^2^) = 0.171	*w* = 1/[σ^2^(*F*_o_^2^) + (0.0734*P*)^2^ + 0.5789*P*] where *P* = (*F*_o_^2^ + 2*F*_c_^2^)/3
*S* = 1.08	(Δ/σ)_max_ = 0.002
8770 reflections	Δρ_max_ = 0.64 e Å^−^^3^
391 parameters	Δρ_min_ = −0.39 e Å^−^^3^
1 restraint	Absolute structure: Flack (1983), 4183 Friedel pairs
Primary atom site location: structure-invariant direct methods	Flack parameter: 0.004 (21)

### Special details

**Table:**

Geometry. All esds (except the esd in the dihedral angle between two l.s. planes) are estimated using the full covariance matrix. The cell esds are taken into account individually in the estimation of esds in distances, angles and torsion angles; correlations between esds in cell parameters are only used when they are defined by crystal symmetry. An approximate (isotropic) treatment of cell esds is used for estimating esds involving l.s. planes.
Refinement. A certain number of restraints were set during the refinement: EADP C1 C2 C3 C4 C5 EADP C36 C37 C38 C39 C35 EADP C22 C23 C21

### Fractional atomic coordinates and isotropic or equivalent isotropic displacement parameters (Å^2^)

**Table:**

	*x*	*y*	*z*	*U*_iso_*/*U*_eq_	
Fe1	−0.16586 (6)	0.33985 (4)	0.47266 (6)	0.05329 (18)	
Fe2	0.50851 (7)	0.00203 (4)	1.22726 (8)	0.0603 (2)	
N1	−0.0020 (8)	0.1299 (6)	1.2572 (6)	0.140 (4)	
N2	−0.2109 (7)	0.2738 (5)	0.9801 (8)	0.117 (2)	
N3	0.3525 (4)	0.1855 (2)	0.9336 (3)	0.0521 (9)	
N4	0.1016 (4)	0.1619 (2)	0.7196 (3)	0.0494 (8)	
C1	−0.3415 (7)	0.3997 (4)	0.3903 (7)	0.0823 (7)	
H1	−0.3587	0.4496	0.4096	0.099*	
C2	−0.2836 (7)	0.3759 (4)	0.2921 (7)	0.0823 (7)	
H2	−0.2548	0.4073	0.2362	0.099*	
C3	−0.2784 (7)	0.2946 (4)	0.2961 (8)	0.0823 (7)	
H3	−0.2462	0.2634	0.2423	0.099*	
C4	−0.3287 (6)	0.2709 (4)	0.3918 (7)	0.0823 (7)	
H4	−0.3362	0.2203	0.4140	0.099*	
C5	−0.3675 (6)	0.3333 (4)	0.4524 (6)	0.0823 (7)	
H5	−0.4040	0.3314	0.5213	0.099*	
C6	−0.0596 (6)	0.3356 (4)	0.6669 (5)	0.0695 (13)	
H6	−0.0963	0.3298	0.7350	0.083*	
C7	−0.0367 (8)	0.4062 (4)	0.6129 (8)	0.097 (3)	
H7	−0.0566	0.4542	0.6385	0.116*	
C8	0.0208 (7)	0.3905 (4)	0.5145 (8)	0.091 (2)	
H8	0.0460	0.4266	0.4632	0.109*	
C9	0.0343 (5)	0.3130 (4)	0.5051 (6)	0.0694 (15)	
H9	0.0707	0.2884	0.4471	0.083*	
C10	−0.0180 (5)	0.2758 (3)	0.6010 (4)	0.0499 (10)	
C11	−0.0209 (5)	0.1914 (3)	0.6200 (5)	0.0563 (11)	
H11A	−0.1007	0.1786	0.6452	0.068*	
H11B	−0.0302	0.1664	0.5371	0.068*	
C12	0.0791 (6)	0.0808 (3)	0.7416 (5)	0.0613 (12)	
H12A	0.1659	0.0574	0.7889	0.074*	
H12B	0.0437	0.0554	0.6575	0.074*	
C13	−0.0177 (5)	0.0698 (3)	0.8166 (6)	0.0618 (13)	
C14	−0.1466 (6)	0.0366 (3)	0.7607 (7)	0.0781 (17)	
H14	−0.1749	0.0219	0.6730	0.094*	
C15	−0.2331 (6)	0.0254 (4)	0.8373 (7)	0.0851 (19)	
H15A	−0.3170	0.0014	0.8005	0.102*	
C16	−0.1963 (6)	0.0492 (4)	0.9657 (7)	0.0804 (17)	
H16	−0.2550	0.0426	1.0152	0.097*	
C17	−0.0707 (6)	0.0830 (4)	1.0190 (6)	0.0722 (15)	
C18	0.0171 (6)	0.0936 (3)	0.9461 (5)	0.0655 (13)	
H18	0.1011	0.1171	0.9847	0.079*	
C19	−0.0319 (7)	0.1082 (5)	1.1527 (7)	0.092 (2)	
C20	0.2301 (5)	0.1777 (3)	0.6915 (4)	0.0537 (11)	
H20	0.2315	0.2326	0.6764	0.064*	
C21	0.2416 (7)	0.1391 (5)	0.5673 (6)	0.0954 (13)	
H21A	0.1593	0.1488	0.4945	0.114*	
H21B	0.2507	0.0846	0.5808	0.114*	
C22	0.3673 (7)	0.1703 (6)	0.5344 (6)	0.0954 (13)	
H22A	0.3737	0.1459	0.4556	0.114*	
H22B	0.3569	0.2246	0.5183	0.114*	
C23	0.4977 (7)	0.1548 (5)	0.6485 (6)	0.0954 (13)	
H23A	0.5113	0.1004	0.6606	0.114*	
H23B	0.5758	0.1759	0.6285	0.114*	
C24	0.4874 (6)	0.1900 (4)	0.7722 (6)	0.0777 (17)	
H24A	0.4805	0.2448	0.7617	0.093*	
H24B	0.5701	0.1788	0.8437	0.093*	
C25	0.3603 (5)	0.1596 (3)	0.8077 (4)	0.0556 (11)	
H25	0.3687	0.1041	0.8130	0.067*	
C26	0.3528 (5)	0.2688 (3)	0.9502 (5)	0.0571 (11)	
H26A	0.3144	0.2930	0.8653	0.068*	
H26B	0.4460	0.2867	0.9885	0.068*	
C27	0.2695 (5)	0.2898 (3)	1.0380 (4)	0.0527 (10)	
C28	0.1312 (5)	0.2788 (3)	0.9918 (5)	0.0616 (12)	
H28	0.0911	0.2585	0.9085	0.074*	
C29	0.0497 (6)	0.2980 (3)	1.0697 (5)	0.0688 (14)	
C30	0.1084 (7)	0.3275 (4)	1.1928 (5)	0.0799 (17)	
H30	0.0539	0.3416	1.2435	0.096*	
C31	0.2463 (8)	0.3362 (5)	1.2407 (6)	0.094 (2)	
H31	0.2867	0.3539	1.3257	0.113*	
C32	0.3277 (6)	0.3185 (3)	1.1620 (5)	0.0689 (15)	
H32	0.4217	0.3263	1.1939	0.083*	
C33	−0.0945 (8)	0.2853 (4)	1.0194 (7)	0.0866 (19)	
C34	0.4532 (5)	0.1489 (3)	1.0455 (4)	0.0566 (11)	
H34A	0.4717	0.1822	1.1211	0.068*	
H34B	0.5379	0.1423	1.0255	0.068*	
C35	0.4078 (6)	0.0744 (4)	1.0789 (7)	0.0799 (8)	
C36	0.4293 (5)	0.0010 (5)	1.0245 (6)	0.0799 (8)	
H36	0.4760	−0.0066	0.9642	0.096*	
C37	0.3679 (6)	−0.0560 (4)	1.0781 (7)	0.0799 (8)	
H37	0.3688	−0.1079	1.0614	0.096*	
C38	0.3064 (6)	−0.0217 (4)	1.1597 (7)	0.0799 (8)	
H38	0.2570	−0.0471	1.2060	0.096*	
C39	0.3300 (6)	0.0607 (4)	1.1635 (7)	0.0799 (8)	
H39	0.2998	0.0966	1.2118	0.096*	
C40	0.6277 (7)	0.0454 (4)	1.4035 (6)	0.0726 (15)	
H40	0.6097	0.0881	1.4464	0.087*	
C41	0.5881 (7)	−0.0292 (4)	1.4178 (7)	0.089 (2)	
H41	0.5370	−0.0447	1.4708	0.106*	
C42	0.6371 (8)	−0.0758 (4)	1.3407 (7)	0.088 (2)	
H42	0.6265	−0.1284	1.3339	0.106*	
C43	0.7059 (7)	−0.0309 (5)	1.2735 (7)	0.087 (2)	
H43	0.7477	−0.0484	1.2139	0.104*	
C44	0.7007 (6)	0.0440 (4)	1.3113 (6)	0.0706 (15)	
H44	0.7380	0.0858	1.2818	0.085*	

### Atomic displacement parameters (Å^2^)

**Table:**

	*U*^11^	*U*^22^	*U*^33^	*U*^12^	*U*^13^	*U*^23^
Fe1	0.0469 (3)	0.0523 (3)	0.0521 (3)	0.0031 (3)	0.0033 (3)	0.0070 (3)
Fe2	0.0492 (4)	0.0593 (4)	0.0643 (4)	0.0012 (3)	0.0063 (3)	0.0189 (3)
N1	0.116 (6)	0.244 (11)	0.061 (4)	−0.045 (6)	0.029 (4)	−0.002 (5)
N2	0.070 (4)	0.130 (6)	0.141 (6)	0.014 (4)	0.021 (4)	−0.009 (5)
N3	0.0495 (19)	0.055 (2)	0.0442 (18)	0.0088 (17)	0.0038 (15)	0.0059 (16)
N4	0.053 (2)	0.0478 (19)	0.0429 (18)	0.0076 (16)	0.0096 (15)	0.0047 (15)
C1	0.0626 (14)	0.0828 (17)	0.0867 (17)	0.0045 (16)	0.0025 (12)	0.0040 (17)
C2	0.0626 (14)	0.0828 (17)	0.0867 (17)	0.0045 (16)	0.0025 (12)	0.0040 (17)
C3	0.0626 (14)	0.0828 (17)	0.0867 (17)	0.0045 (16)	0.0025 (12)	0.0040 (17)
C4	0.0626 (14)	0.0828 (17)	0.0867 (17)	0.0045 (16)	0.0025 (12)	0.0040 (17)
C5	0.0626 (14)	0.0828 (17)	0.0867 (17)	0.0045 (16)	0.0025 (12)	0.0040 (17)
C6	0.077 (3)	0.069 (3)	0.050 (2)	0.020 (3)	0.002 (2)	−0.007 (3)
C7	0.090 (5)	0.058 (4)	0.106 (6)	0.003 (3)	−0.020 (4)	−0.009 (4)
C8	0.061 (4)	0.072 (4)	0.110 (6)	−0.013 (3)	−0.016 (4)	0.034 (4)
C9	0.052 (3)	0.082 (4)	0.070 (3)	0.009 (2)	0.013 (2)	0.028 (3)
C10	0.046 (2)	0.056 (3)	0.039 (2)	0.008 (2)	0.0001 (17)	0.0040 (19)
C11	0.052 (2)	0.062 (3)	0.048 (2)	0.005 (2)	0.006 (2)	−0.001 (2)
C12	0.066 (3)	0.054 (3)	0.060 (3)	0.008 (2)	0.014 (2)	−0.001 (2)
C13	0.056 (3)	0.047 (3)	0.070 (3)	0.005 (2)	0.002 (2)	0.007 (2)
C14	0.076 (4)	0.057 (3)	0.084 (4)	0.001 (3)	0.002 (3)	−0.006 (3)
C15	0.060 (3)	0.080 (4)	0.105 (5)	−0.019 (3)	0.011 (3)	−0.007 (4)
C16	0.061 (3)	0.072 (4)	0.104 (5)	−0.004 (3)	0.020 (3)	0.018 (3)
C17	0.062 (3)	0.073 (3)	0.072 (3)	−0.007 (3)	0.008 (3)	0.020 (3)
C18	0.055 (3)	0.077 (3)	0.057 (3)	−0.004 (3)	0.006 (2)	0.007 (2)
C19	0.070 (4)	0.134 (6)	0.068 (4)	−0.019 (4)	0.017 (3)	0.020 (4)
C20	0.053 (2)	0.062 (3)	0.043 (2)	0.012 (2)	0.0108 (19)	0.006 (2)
C21	0.087 (2)	0.143 (4)	0.063 (2)	0.035 (3)	0.0324 (18)	0.006 (2)
C22	0.087 (2)	0.143 (4)	0.063 (2)	0.035 (3)	0.0324 (18)	0.006 (2)
C23	0.087 (2)	0.143 (4)	0.063 (2)	0.035 (3)	0.0324 (18)	0.006 (2)
C24	0.055 (3)	0.110 (5)	0.072 (4)	0.022 (3)	0.027 (3)	0.019 (3)
C25	0.048 (2)	0.066 (3)	0.052 (2)	0.017 (2)	0.0137 (19)	0.005 (2)
C26	0.057 (3)	0.054 (3)	0.060 (3)	0.001 (2)	0.018 (2)	0.004 (2)
C27	0.057 (3)	0.047 (2)	0.047 (2)	0.009 (2)	0.0076 (19)	0.0108 (18)
C28	0.058 (3)	0.073 (3)	0.047 (2)	0.014 (2)	0.007 (2)	0.005 (2)
C29	0.070 (3)	0.071 (3)	0.062 (3)	0.009 (3)	0.017 (3)	−0.003 (3)
C30	0.102 (4)	0.087 (4)	0.054 (3)	0.016 (4)	0.029 (3)	−0.005 (3)
C31	0.128 (6)	0.087 (4)	0.050 (3)	0.002 (5)	0.002 (3)	−0.017 (3)
C32	0.072 (3)	0.059 (3)	0.059 (3)	0.000 (2)	−0.004 (3)	−0.004 (2)
C33	0.082 (4)	0.090 (5)	0.092 (5)	0.022 (4)	0.033 (4)	0.003 (4)
C34	0.058 (3)	0.054 (3)	0.049 (2)	0.008 (2)	0.005 (2)	0.004 (2)
C35	0.0567 (14)	0.0802 (17)	0.0893 (19)	−0.0014 (13)	0.0037 (12)	0.0219 (16)
C36	0.0567 (14)	0.0802 (17)	0.0893 (19)	−0.0014 (13)	0.0037 (12)	0.0219 (16)
C37	0.0567 (14)	0.0802 (17)	0.0893 (19)	−0.0014 (13)	0.0037 (12)	0.0219 (16)
C38	0.0567 (14)	0.0802 (17)	0.0893 (19)	−0.0014 (13)	0.0037 (12)	0.0219 (16)
C39	0.0567 (14)	0.0802 (17)	0.0893 (19)	−0.0014 (13)	0.0037 (12)	0.0219 (16)
C40	0.081 (4)	0.074 (4)	0.062 (3)	−0.003 (3)	0.020 (3)	0.009 (3)
C41	0.082 (4)	0.104 (5)	0.070 (4)	−0.011 (4)	0.010 (3)	0.048 (4)
C42	0.091 (5)	0.062 (3)	0.083 (4)	0.021 (3)	−0.015 (4)	0.023 (3)
C43	0.067 (4)	0.107 (5)	0.079 (4)	0.035 (4)	0.014 (3)	0.012 (4)
C44	0.049 (3)	0.089 (4)	0.067 (3)	−0.005 (3)	0.008 (2)	0.015 (3)

### Geometric parameters (Å, °)

**Table:**

Fe1—C5	2.023 (6)	C15—H15A	0.9300
Fe1—C6	2.029 (5)	C16—C17	1.374 (8)
Fe1—C4	2.029 (7)	C16—H16	0.9300
Fe1—C7	2.033 (7)	C17—C18	1.383 (8)
Fe1—C9	2.035 (5)	C17—C19	1.435 (10)
Fe1—C8	2.036 (7)	C18—H18	0.9300
Fe1—C1	2.038 (7)	C20—C21	1.535 (8)
Fe1—C2	2.038 (7)	C20—C25	1.550 (6)
Fe1—C10	2.041 (4)	C20—H20	0.9800
Fe1—C3	2.047 (7)	C21—C22	1.545 (10)
Fe2—C43	2.019 (6)	C21—H21A	0.9700
Fe2—C38	2.020 (6)	C21—H21B	0.9700
Fe2—C42	2.023 (5)	C22—C23	1.530 (9)
Fe2—C41	2.026 (6)	C22—H22A	0.9700
Fe2—C39	2.030 (6)	C22—H22B	0.9700
Fe2—C44	2.037 (6)	C23—C24	1.499 (9)
Fe2—C35	2.046 (6)	C23—H23A	0.9700
Fe2—C40	2.051 (7)	C23—H23B	0.9700
Fe2—C37	2.059 (7)	C24—C25	1.569 (7)
Fe2—C36	2.072 (6)	C24—H24A	0.9700
N1—C19	1.133 (9)	C24—H24B	0.9700
N2—C33	1.156 (9)	C25—H25	0.9800
N3—C25	1.453 (6)	C26—C27	1.508 (7)
N3—C34	1.465 (5)	C26—H26A	0.9700
N3—C26	1.475 (6)	C26—H26B	0.9700
N4—C11	1.468 (5)	C27—C28	1.366 (7)
N4—C12	1.474 (6)	C27—C32	1.373 (7)
N4—C20	1.474 (6)	C28—C29	1.401 (8)
C1—C5	1.412 (10)	C28—H28	0.9300
C1—C2	1.429 (11)	C29—C30	1.372 (8)
C1—H1	0.9300	C29—C33	1.429 (10)
C2—C3	1.430 (9)	C30—C31	1.358 (10)
C2—H2	0.9300	C30—H30	0.9300
C3—C4	1.355 (10)	C31—C32	1.401 (9)
C3—H3	0.9300	C31—H31	0.9300
C4—C5	1.397 (10)	C32—H32	0.9300
C4—H4	0.9300	C34—C35	1.473 (8)
C5—H5	0.9300	C34—H34A	0.9700
C6—C10	1.408 (7)	C34—H34B	0.9700
C6—C7	1.420 (10)	C35—C39	1.410 (10)
C6—H6	0.9300	C35—C36	1.461 (11)
C7—C8	1.393 (11)	C36—C37	1.402 (9)
C7—H7	0.9300	C36—H36	0.9300
C8—C9	1.375 (10)	C37—C38	1.372 (10)
C8—H8	0.9300	C37—H37	0.9300
C9—C10	1.460 (7)	C38—C39	1.467 (10)
C9—H9	0.9300	C38—H38	0.9300
C10—C11	1.498 (7)	C39—H39	0.9300
C11—H11A	0.9700	C40—C41	1.395 (9)
C11—H11B	0.9700	C40—C44	1.420 (9)
C12—C13	1.479 (8)	C40—H40	0.9300
C12—H12A	0.9700	C41—C42	1.370 (11)
C12—H12B	0.9700	C41—H41	0.9300
C13—C18	1.388 (8)	C42—C43	1.403 (11)
C13—C14	1.402 (8)	C42—H42	0.9300
C14—C15	1.405 (9)	C43—C44	1.384 (10)
C14—H14	0.9300	C43—H43	0.9300
C15—C16	1.376 (10)	C44—H44	0.9300
			
C5—Fe1—C6	108.1 (3)	N4—C11—H11A	108.9
C5—Fe1—C4	40.4 (3)	C10—C11—H11A	108.9
C6—Fe1—C4	121.5 (3)	N4—C11—H11B	108.9
C5—Fe1—C7	121.5 (3)	C10—C11—H11B	108.9
C6—Fe1—C7	40.9 (3)	H11A—C11—H11B	107.7
C4—Fe1—C7	156.8 (4)	N4—C12—C13	112.4 (4)
C5—Fe1—C9	162.9 (3)	N4—C12—H12A	109.1
C6—Fe1—C9	68.1 (2)	C13—C12—H12A	109.1
C4—Fe1—C9	126.2 (3)	N4—C12—H12B	109.1
C7—Fe1—C9	67.4 (3)	C13—C12—H12B	109.1
C5—Fe1—C8	156.2 (3)	H12A—C12—H12B	107.9
C6—Fe1—C8	67.9 (3)	C18—C13—C14	117.7 (6)
C4—Fe1—C8	162.0 (4)	C18—C13—C12	120.2 (5)
C7—Fe1—C8	40.1 (3)	C14—C13—C12	122.1 (6)
C9—Fe1—C8	39.5 (3)	C13—C14—C15	119.9 (6)
C5—Fe1—C1	40.7 (3)	C13—C14—H14	120.1
C6—Fe1—C1	125.6 (3)	C15—C14—H14	120.1
C4—Fe1—C1	67.8 (3)	C16—C15—C14	121.4 (6)
C7—Fe1—C1	108.0 (3)	C16—C15—H15A	119.3
C9—Fe1—C1	155.1 (3)	C14—C15—H15A	119.3
C8—Fe1—C1	121.2 (3)	C17—C16—C15	118.3 (7)
C5—Fe1—C2	68.5 (3)	C17—C16—H16	120.8
C6—Fe1—C2	163.1 (3)	C15—C16—H16	120.8
C4—Fe1—C2	67.3 (3)	C16—C17—C18	121.4 (6)
C7—Fe1—C2	125.5 (3)	C16—C17—C19	118.6 (6)
C9—Fe1—C2	120.1 (3)	C18—C17—C19	120.0 (6)
C8—Fe1—C2	108.1 (3)	C17—C18—C13	121.3 (5)
C1—Fe1—C2	41.1 (3)	C17—C18—H18	119.4
C5—Fe1—C10	124.3 (2)	C13—C18—H18	119.4
C6—Fe1—C10	40.5 (2)	N1—C19—C17	178.4 (10)
C4—Fe1—C10	107.4 (3)	N4—C20—C21	114.6 (5)
C7—Fe1—C10	69.0 (2)	N4—C20—C25	113.7 (4)
C9—Fe1—C10	42.0 (2)	C21—C20—C25	109.0 (4)
C8—Fe1—C10	68.9 (2)	N4—C20—H20	106.3
C1—Fe1—C10	161.7 (3)	C21—C20—H20	106.3
C2—Fe1—C10	155.3 (3)	C25—C20—H20	106.3
C5—Fe1—C3	67.4 (3)	C20—C21—C22	110.1 (6)
C6—Fe1—C3	154.6 (3)	C20—C21—H21A	109.6
C4—Fe1—C3	38.8 (3)	C22—C21—H21A	109.6
C7—Fe1—C3	163.1 (3)	C20—C21—H21B	109.6
C9—Fe1—C3	108.4 (3)	C22—C21—H21B	109.6
C8—Fe1—C3	126.4 (4)	H21A—C21—H21B	108.2
C1—Fe1—C3	68.4 (3)	C23—C22—C21	110.0 (6)
C2—Fe1—C3	41.0 (2)	C23—C22—H22A	109.7
C10—Fe1—C3	119.7 (2)	C21—C22—H22A	109.7
C43—Fe2—C38	150.8 (3)	C23—C22—H22B	109.7
C43—Fe2—C42	40.6 (3)	C21—C22—H22B	109.7
C38—Fe2—C42	118.8 (3)	H22A—C22—H22B	108.2
C43—Fe2—C41	67.6 (3)	C24—C23—C22	110.3 (5)
C38—Fe2—C41	110.5 (3)	C24—C23—H23A	109.6
C42—Fe2—C41	39.6 (3)	C22—C23—H23A	109.6
C43—Fe2—C39	164.4 (3)	C24—C23—H23B	109.6
C38—Fe2—C39	42.5 (3)	C22—C23—H23B	109.6
C42—Fe2—C39	153.9 (3)	H23A—C23—H23B	108.1
C41—Fe2—C39	120.9 (3)	C23—C24—C25	111.9 (6)
C43—Fe2—C44	39.9 (3)	C23—C24—H24A	109.2
C38—Fe2—C44	168.8 (3)	C25—C24—H24A	109.2
C42—Fe2—C44	67.6 (3)	C23—C24—H24B	109.2
C41—Fe2—C44	67.9 (3)	C25—C24—H24B	109.2
C39—Fe2—C44	128.0 (3)	H24A—C24—H24B	107.9
C43—Fe2—C35	126.4 (3)	N3—C25—C20	113.4 (4)
C38—Fe2—C35	68.7 (2)	N3—C25—C24	114.6 (4)
C42—Fe2—C35	164.5 (3)	C20—C25—C24	108.1 (4)
C41—Fe2—C35	154.0 (3)	N3—C25—H25	106.7
C39—Fe2—C35	40.5 (3)	C20—C25—H25	106.7
C44—Fe2—C35	107.6 (2)	C24—C25—H25	106.7
C43—Fe2—C40	67.4 (3)	N3—C26—C27	109.8 (4)
C38—Fe2—C40	131.1 (3)	N3—C26—H26A	109.7
C42—Fe2—C40	66.9 (3)	C27—C26—H26A	109.7
C41—Fe2—C40	40.0 (3)	N3—C26—H26B	109.7
C39—Fe2—C40	109.7 (3)	C27—C26—H26B	109.7
C44—Fe2—C40	40.6 (3)	H26A—C26—H26B	108.2
C35—Fe2—C40	119.8 (3)	C28—C27—C32	119.4 (5)
C43—Fe2—C37	117.1 (3)	C28—C27—C26	118.2 (4)
C38—Fe2—C37	39.3 (3)	C32—C27—C26	122.5 (5)
C42—Fe2—C37	107.5 (3)	C27—C28—C29	120.1 (5)
C41—Fe2—C37	127.8 (3)	C27—C28—H28	119.9
C39—Fe2—C37	69.5 (3)	C29—C28—H28	119.9
C44—Fe2—C37	150.5 (3)	C30—C29—C28	120.2 (6)
C35—Fe2—C37	69.0 (3)	C30—C29—C33	120.8 (6)
C40—Fe2—C37	166.3 (3)	C28—C29—C33	119.0 (5)
C43—Fe2—C36	106.7 (3)	C31—C30—C29	119.8 (6)
C38—Fe2—C36	66.5 (3)	C31—C30—H30	120.1
C42—Fe2—C36	126.4 (3)	C29—C30—H30	120.1
C41—Fe2—C36	163.7 (3)	C30—C31—C32	120.0 (5)
C39—Fe2—C36	68.6 (3)	C30—C31—H31	120.0
C44—Fe2—C36	118.3 (2)	C32—C31—H31	120.0
C35—Fe2—C36	41.6 (3)	C27—C32—C31	120.4 (5)
C40—Fe2—C36	153.7 (2)	C27—C32—H32	119.8
C37—Fe2—C36	39.7 (3)	C31—C32—H32	119.8
C25—N3—C34	113.2 (4)	N2—C33—C29	178.7 (8)
C25—N3—C26	115.1 (4)	N3—C34—C35	113.2 (4)
C34—N3—C26	111.3 (4)	N3—C34—H34A	108.9
C11—N4—C12	108.4 (4)	C35—C34—H34A	108.9
C11—N4—C20	113.9 (3)	N3—C34—H34B	108.9
C12—N4—C20	114.3 (4)	C35—C34—H34B	108.9
C5—C1—C2	107.0 (7)	H34A—C34—H34B	107.8
C5—C1—Fe1	69.1 (4)	C39—C35—C36	107.3 (6)
C2—C1—Fe1	69.5 (4)	C39—C35—C34	126.7 (7)
C5—C1—H1	126.5	C36—C35—C34	126.0 (6)
C2—C1—H1	126.5	C39—C35—Fe2	69.1 (4)
Fe1—C1—H1	126.5	C36—C35—Fe2	70.1 (3)
C1—C2—C3	106.9 (8)	C34—C35—Fe2	128.4 (4)
C1—C2—Fe1	69.5 (4)	C37—C36—C35	108.7 (6)
C3—C2—Fe1	69.8 (5)	C37—C36—Fe2	69.7 (4)
C1—C2—H2	126.5	C35—C36—Fe2	68.3 (4)
C3—C2—H2	126.5	C37—C36—H36	125.7
Fe1—C2—H2	125.7	C35—C36—H36	125.6
C4—C3—C2	108.1 (8)	Fe2—C36—H36	128.0
C4—C3—Fe1	69.9 (4)	C38—C37—C36	108.0 (7)
C2—C3—Fe1	69.2 (5)	C38—C37—Fe2	68.8 (4)
C4—C3—H3	126.0	C36—C37—Fe2	70.6 (4)
C2—C3—H3	126.0	C38—C37—H37	126.0
Fe1—C3—H3	126.6	C36—C37—H37	126.0
C3—C4—C5	110.3 (7)	Fe2—C37—H37	126.1
C3—C4—Fe1	71.3 (4)	C37—C38—C39	110.2 (7)
C5—C4—Fe1	69.6 (4)	C37—C38—Fe2	71.9 (4)
C3—C4—H4	124.8	C39—C38—Fe2	69.1 (4)
C5—C4—H4	124.8	C37—C38—H38	124.9
Fe1—C4—H4	125.9	C39—C38—H38	124.9
C4—C5—C1	107.7 (6)	Fe2—C38—H38	125.7
C4—C5—Fe1	70.1 (4)	C35—C39—C38	105.8 (7)
C1—C5—Fe1	70.2 (4)	C35—C39—Fe2	70.4 (4)
C4—C5—H5	126.2	C38—C39—Fe2	68.4 (4)
C1—C5—H5	126.2	C35—C39—H39	127.1
Fe1—C5—H5	125.1	C38—C39—H39	127.1
C10—C6—C7	109.3 (6)	Fe2—C39—H39	125.7
C10—C6—Fe1	70.2 (3)	C41—C40—C44	107.4 (7)
C7—C6—Fe1	69.7 (4)	C41—C40—Fe2	69.1 (4)
C10—C6—H6	125.3	C44—C40—Fe2	69.2 (4)
C7—C6—H6	125.3	C41—C40—H40	126.3
Fe1—C6—H6	126.4	C44—C40—H40	126.3
C8—C7—C6	107.6 (6)	Fe2—C40—H40	127.0
C8—C7—Fe1	70.1 (4)	C42—C41—C40	108.6 (6)
C6—C7—Fe1	69.4 (3)	C42—C41—Fe2	70.1 (4)
C8—C7—H7	126.2	C40—C41—Fe2	70.9 (4)
C6—C7—H7	126.2	C42—C41—H41	125.7
Fe1—C7—H7	125.9	C40—C41—H41	125.7
C9—C8—C7	109.2 (7)	Fe2—C41—H41	124.9
C9—C8—Fe1	70.2 (4)	C41—C42—C43	108.5 (6)
C7—C8—Fe1	69.9 (4)	C41—C42—Fe2	70.4 (3)
C9—C8—H8	125.4	C43—C42—Fe2	69.5 (3)
C7—C8—H8	125.4	C41—C42—H42	125.8
Fe1—C8—H8	126.1	C43—C42—H42	125.8
C8—C9—C10	108.9 (7)	Fe2—C42—H42	125.9
C8—C9—Fe1	70.3 (4)	C44—C43—C42	108.2 (7)
C10—C9—Fe1	69.2 (3)	C44—C43—Fe2	70.8 (4)
C8—C9—H9	125.6	C42—C43—Fe2	69.8 (4)
C10—C9—H9	125.6	C44—C43—H43	125.9
Fe1—C9—H9	126.5	C42—C43—H43	125.9
C6—C10—C9	105.0 (5)	Fe2—C43—H43	125.1
C6—C10—C11	130.5 (5)	C43—C44—C40	107.4 (6)
C9—C10—C11	124.6 (5)	C43—C44—Fe2	69.3 (4)
C6—C10—Fe1	69.3 (3)	C40—C44—Fe2	70.2 (4)
C9—C10—Fe1	68.8 (3)	C43—C44—H44	126.3
C11—C10—Fe1	126.5 (3)	C40—C44—H44	126.3
N4—C11—C10	113.6 (4)	Fe2—C44—H44	125.7
			
C6—Fe1—C1—C5	−75.7 (5)	C26—N3—C25—C20	−68.4 (6)
C4—Fe1—C1—C5	38.0 (4)	C34—N3—C25—C24	−73.0 (5)
C7—Fe1—C1—C5	−117.6 (5)	C26—N3—C25—C24	56.5 (5)
C9—Fe1—C1—C5	167.4 (6)	N4—C20—C25—N3	−43.6 (6)
C8—Fe1—C1—C5	−159.5 (5)	C21—C20—C25—N3	−172.8 (5)
C2—Fe1—C1—C5	118.6 (6)	N4—C20—C25—C24	−171.8 (4)
C10—Fe1—C1—C5	−40.1 (10)	C21—C20—C25—C24	59.0 (6)
C3—Fe1—C1—C5	80.0 (5)	C23—C24—C25—N3	173.9 (5)
C5—Fe1—C1—C2	−118.6 (6)	C23—C24—C25—C20	−58.5 (7)
C6—Fe1—C1—C2	165.7 (4)	C25—N3—C26—C27	148.3 (4)
C4—Fe1—C1—C2	−80.5 (5)	C34—N3—C26—C27	−81.2 (5)
C7—Fe1—C1—C2	123.8 (5)	N3—C26—C27—C28	−68.3 (5)
C9—Fe1—C1—C2	48.9 (8)	N3—C26—C27—C32	111.0 (5)
C8—Fe1—C1—C2	81.9 (5)	C32—C27—C28—C29	1.1 (8)
C10—Fe1—C1—C2	−158.7 (7)	C26—C27—C28—C29	−179.6 (5)
C3—Fe1—C1—C2	−38.5 (4)	C27—C28—C29—C30	−0.3 (9)
C5—C1—C2—C3	1.0 (7)	C27—C28—C29—C33	−179.5 (5)
Fe1—C1—C2—C3	60.1 (5)	C28—C29—C30—C31	−1.8 (10)
C5—C1—C2—Fe1	−59.1 (4)	C33—C29—C30—C31	177.4 (7)
C5—Fe1—C2—C1	38.0 (4)	C29—C30—C31—C32	3.1 (11)
C6—Fe1—C2—C1	−43.4 (12)	C28—C27—C32—C31	0.2 (8)
C4—Fe1—C2—C1	81.7 (5)	C26—C27—C32—C31	−179.1 (5)
C7—Fe1—C2—C1	−76.0 (6)	C30—C31—C32—C27	−2.3 (10)
C9—Fe1—C2—C1	−158.5 (4)	C25—N3—C34—C35	−83.8 (6)
C8—Fe1—C2—C1	−116.9 (5)	C26—N3—C34—C35	144.7 (5)
C10—Fe1—C2—C1	164.2 (5)	N3—C34—C35—C39	−87.6 (7)
C3—Fe1—C2—C1	117.9 (7)	N3—C34—C35—C36	88.8 (6)
C5—Fe1—C2—C3	−79.9 (5)	N3—C34—C35—Fe2	−178.9 (4)
C6—Fe1—C2—C3	−161.3 (8)	C43—Fe2—C35—C39	−168.5 (4)
C4—Fe1—C2—C3	−36.2 (5)	C38—Fe2—C35—C39	40.3 (4)
C7—Fe1—C2—C3	166.0 (5)	C42—Fe2—C35—C39	162.2 (10)
C9—Fe1—C2—C3	83.6 (5)	C41—Fe2—C35—C39	−53.0 (8)
C8—Fe1—C2—C3	125.1 (5)	C44—Fe2—C35—C39	−128.5 (4)
C1—Fe1—C2—C3	−117.9 (7)	C40—Fe2—C35—C39	−85.9 (5)
C10—Fe1—C2—C3	46.3 (9)	C37—Fe2—C35—C39	82.6 (4)
C1—C2—C3—C4	−0.6 (8)	C36—Fe2—C35—C39	118.4 (5)
Fe1—C2—C3—C4	59.2 (5)	C43—Fe2—C35—C36	73.1 (5)
C1—C2—C3—Fe1	−59.9 (5)	C38—Fe2—C35—C36	−78.1 (4)
C5—Fe1—C3—C4	−36.9 (4)	C42—Fe2—C35—C36	43.8 (12)
C6—Fe1—C3—C4	47.9 (8)	C41—Fe2—C35—C36	−171.4 (5)
C7—Fe1—C3—C4	−162.0 (10)	C39—Fe2—C35—C36	−118.4 (5)
C9—Fe1—C3—C4	125.4 (4)	C44—Fe2—C35—C36	113.1 (4)
C8—Fe1—C3—C4	165.5 (4)	C40—Fe2—C35—C36	155.7 (3)
C1—Fe1—C3—C4	−80.9 (4)	C37—Fe2—C35—C36	−35.9 (4)
C2—Fe1—C3—C4	−119.5 (7)	C43—Fe2—C35—C34	−47.6 (8)
C10—Fe1—C3—C4	80.8 (5)	C38—Fe2—C35—C34	161.2 (7)
C5—Fe1—C3—C2	82.7 (5)	C42—Fe2—C35—C34	−76.9 (13)
C6—Fe1—C3—C2	167.5 (5)	C41—Fe2—C35—C34	67.9 (9)
C4—Fe1—C3—C2	119.5 (7)	C39—Fe2—C35—C34	120.9 (8)
C7—Fe1—C3—C2	−42.4 (13)	C44—Fe2—C35—C34	−7.6 (7)
C9—Fe1—C3—C2	−115.0 (5)	C40—Fe2—C35—C34	35.1 (7)
C8—Fe1—C3—C2	−75.0 (6)	C37—Fe2—C35—C34	−156.5 (7)
C1—Fe1—C3—C2	38.6 (5)	C36—Fe2—C35—C34	−120.7 (8)
C10—Fe1—C3—C2	−159.6 (4)	C39—C35—C36—C37	−1.4 (6)
C2—C3—C4—C5	0.0 (8)	C34—C35—C36—C37	−178.4 (5)
Fe1—C3—C4—C5	58.8 (4)	Fe2—C35—C36—C37	58.0 (4)
C2—C3—C4—Fe1	−58.8 (5)	C39—C35—C36—Fe2	−59.4 (4)
C5—Fe1—C4—C3	121.1 (6)	C34—C35—C36—Fe2	123.6 (6)
C6—Fe1—C4—C3	−158.0 (4)	C43—Fe2—C36—C37	112.5 (5)
C7—Fe1—C4—C3	166.8 (6)	C38—Fe2—C36—C37	−37.1 (4)
C9—Fe1—C4—C3	−73.4 (5)	C42—Fe2—C36—C37	72.2 (5)
C8—Fe1—C4—C3	−40.6 (11)	C41—Fe2—C36—C37	45.5 (11)
C1—Fe1—C4—C3	82.8 (5)	C39—Fe2—C36—C37	−83.2 (4)
C2—Fe1—C4—C3	38.2 (4)	C44—Fe2—C36—C37	154.1 (4)
C10—Fe1—C4—C3	−116.0 (4)	C35—Fe2—C36—C37	−121.0 (5)
C6—Fe1—C4—C5	80.8 (5)	C40—Fe2—C36—C37	−174.7 (6)
C7—Fe1—C4—C5	45.7 (9)	C43—Fe2—C36—C35	−126.4 (4)
C9—Fe1—C4—C5	165.5 (4)	C38—Fe2—C36—C35	83.9 (4)
C8—Fe1—C4—C5	−161.8 (8)	C42—Fe2—C36—C35	−166.7 (4)
C1—Fe1—C4—C5	−38.3 (4)	C41—Fe2—C36—C35	166.5 (9)
C2—Fe1—C4—C5	−82.9 (4)	C39—Fe2—C36—C35	37.8 (4)
C10—Fe1—C4—C5	122.9 (4)	C44—Fe2—C36—C35	−84.9 (4)
C3—Fe1—C4—C5	−121.1 (6)	C40—Fe2—C36—C35	−53.7 (7)
C3—C4—C5—C1	0.6 (7)	C37—Fe2—C36—C35	121.0 (5)
Fe1—C4—C5—C1	60.5 (4)	C35—C36—C37—C38	1.7 (7)
C3—C4—C5—Fe1	−59.8 (5)	Fe2—C36—C37—C38	58.9 (4)
C2—C1—C5—C4	−1.0 (6)	C35—C36—C37—Fe2	−57.2 (4)
Fe1—C1—C5—C4	−60.4 (4)	C43—Fe2—C37—C38	157.2 (4)
C2—C1—C5—Fe1	59.4 (4)	C42—Fe2—C37—C38	114.3 (5)
C6—Fe1—C5—C4	−117.7 (4)	C41—Fe2—C37—C38	75.5 (5)
C7—Fe1—C5—C4	−160.7 (4)	C39—Fe2—C37—C38	−38.2 (4)
C9—Fe1—C5—C4	−43.5 (10)	C44—Fe2—C37—C38	−170.6 (5)
C8—Fe1—C5—C4	166.1 (7)	C35—Fe2—C37—C38	−81.6 (4)
C1—Fe1—C5—C4	118.2 (6)	C40—Fe2—C37—C38	50.9 (13)
C2—Fe1—C5—C4	79.9 (4)	C36—Fe2—C37—C38	−119.1 (6)
C10—Fe1—C5—C4	−76.0 (5)	C43—Fe2—C37—C36	−83.6 (5)
C3—Fe1—C5—C4	35.5 (4)	C38—Fe2—C37—C36	119.1 (6)
C6—Fe1—C5—C1	124.0 (4)	C42—Fe2—C37—C36	−126.5 (5)
C4—Fe1—C5—C1	−118.2 (6)	C41—Fe2—C37—C36	−165.3 (5)
C7—Fe1—C5—C1	81.1 (5)	C39—Fe2—C37—C36	80.9 (4)
C9—Fe1—C5—C1	−161.8 (8)	C44—Fe2—C37—C36	−51.4 (7)
C8—Fe1—C5—C1	47.9 (9)	C35—Fe2—C37—C36	37.5 (4)
C2—Fe1—C5—C1	−38.3 (4)	C40—Fe2—C37—C36	170.1 (11)
C10—Fe1—C5—C1	165.8 (4)	C36—C37—C38—C39	−1.4 (7)
C3—Fe1—C5—C1	−82.7 (5)	Fe2—C37—C38—C39	58.7 (4)
C5—Fe1—C6—C10	122.0 (4)	C36—C37—C38—Fe2	−60.0 (4)
C4—Fe1—C6—C10	79.7 (4)	C43—Fe2—C38—C37	−44.9 (7)
C7—Fe1—C6—C10	−120.5 (6)	C42—Fe2—C38—C37	−82.5 (5)
C9—Fe1—C6—C10	−40.3 (3)	C41—Fe2—C38—C37	−125.3 (4)
C8—Fe1—C6—C10	−83.0 (4)	C39—Fe2—C38—C37	120.9 (6)
C1—Fe1—C6—C10	163.7 (4)	C44—Fe2—C38—C37	155.4 (12)
C2—Fe1—C6—C10	−162.6 (9)	C35—Fe2—C38—C37	82.5 (4)
C3—Fe1—C6—C10	46.6 (8)	C40—Fe2—C38—C37	−165.9 (4)
C5—Fe1—C6—C7	−117.4 (5)	C36—Fe2—C38—C37	37.4 (4)
C4—Fe1—C6—C7	−159.7 (5)	C43—Fe2—C38—C39	−165.8 (5)
C9—Fe1—C6—C7	80.3 (5)	C42—Fe2—C38—C39	156.6 (5)
C8—Fe1—C6—C7	37.5 (4)	C41—Fe2—C38—C39	113.8 (5)
C1—Fe1—C6—C7	−75.8 (5)	C44—Fe2—C38—C39	34.5 (16)
C2—Fe1—C6—C7	−42.1 (12)	C35—Fe2—C38—C39	−38.4 (4)
C10—Fe1—C6—C7	120.5 (6)	C40—Fe2—C38—C39	73.2 (5)
C3—Fe1—C6—C7	167.2 (7)	C37—Fe2—C38—C39	−120.9 (6)
C10—C6—C7—C8	−0.7 (7)	C36—Fe2—C38—C39	−83.5 (5)
Fe1—C6—C7—C8	−59.9 (5)	C36—C35—C39—C38	0.5 (6)
C10—C6—C7—Fe1	59.2 (4)	C34—C35—C39—C38	177.5 (5)
C5—Fe1—C7—C8	−159.9 (4)	Fe2—C35—C39—C38	−59.5 (4)
C6—Fe1—C7—C8	118.7 (6)	C36—C35—C39—Fe2	60.0 (4)
C4—Fe1—C7—C8	167.2 (6)	C34—C35—C39—Fe2	−123.0 (6)
C9—Fe1—C7—C8	36.5 (4)	C37—C38—C39—C35	0.5 (7)
C1—Fe1—C7—C8	−117.3 (4)	Fe2—C38—C39—C35	60.8 (4)
C2—Fe1—C7—C8	−75.2 (5)	C37—C38—C39—Fe2	−60.3 (4)
C10—Fe1—C7—C8	81.9 (4)	C43—Fe2—C39—C35	36.6 (13)
C3—Fe1—C7—C8	−42.3 (12)	C38—Fe2—C39—C35	−116.9 (6)
C5—Fe1—C7—C6	81.4 (5)	C42—Fe2—C39—C35	−169.3 (6)
C4—Fe1—C7—C6	48.5 (9)	C41—Fe2—C39—C35	156.0 (4)
C9—Fe1—C7—C6	−82.2 (4)	C44—Fe2—C39—C35	71.1 (5)
C8—Fe1—C7—C6	−118.7 (6)	C40—Fe2—C39—C35	113.2 (4)
C1—Fe1—C7—C6	124.0 (4)	C37—Fe2—C39—C35	−81.4 (4)
C2—Fe1—C7—C6	166.1 (4)	C36—Fe2—C39—C35	−38.8 (4)
C10—Fe1—C7—C6	−36.8 (3)	C43—Fe2—C39—C38	153.5 (10)
C3—Fe1—C7—C6	−160.9 (9)	C42—Fe2—C39—C38	−52.4 (9)
C6—C7—C8—C9	0.1 (8)	C41—Fe2—C39—C38	−87.1 (5)
Fe1—C7—C8—C9	−59.4 (5)	C44—Fe2—C39—C38	−172.0 (4)
C6—C7—C8—Fe1	59.5 (4)	C35—Fe2—C39—C38	116.9 (6)
C5—Fe1—C8—C9	166.8 (6)	C40—Fe2—C39—C38	−130.0 (4)
C6—Fe1—C8—C9	81.9 (4)	C37—Fe2—C39—C38	35.5 (4)
C4—Fe1—C8—C9	−43.3 (11)	C36—Fe2—C39—C38	78.1 (5)
C7—Fe1—C8—C9	120.3 (6)	C43—Fe2—C40—C41	−81.6 (5)
C1—Fe1—C8—C9	−158.8 (4)	C38—Fe2—C40—C41	71.5 (6)
C2—Fe1—C8—C9	−115.6 (4)	C42—Fe2—C40—C41	−37.3 (5)
C10—Fe1—C8—C9	38.3 (4)	C39—Fe2—C40—C41	114.9 (5)
C3—Fe1—C8—C9	−73.8 (5)	C44—Fe2—C40—C41	−119.2 (6)
C5—Fe1—C8—C7	46.5 (9)	C35—Fe2—C40—C41	158.3 (5)
C6—Fe1—C8—C7	−38.3 (4)	C37—Fe2—C40—C41	30.7 (14)
C4—Fe1—C8—C7	−163.6 (8)	C36—Fe2—C40—C41	−163.7 (6)
C9—Fe1—C8—C7	−120.3 (6)	C43—Fe2—C40—C44	37.7 (4)
C1—Fe1—C8—C7	80.9 (5)	C38—Fe2—C40—C44	−169.3 (4)
C2—Fe1—C8—C7	124.1 (4)	C42—Fe2—C40—C44	81.9 (5)
C10—Fe1—C8—C7	−82.0 (4)	C41—Fe2—C40—C44	119.2 (6)
C3—Fe1—C8—C7	165.9 (4)	C39—Fe2—C40—C44	−125.9 (4)
C7—C8—C9—C10	0.6 (7)	C35—Fe2—C40—C44	−82.4 (5)
Fe1—C8—C9—C10	−58.6 (4)	C37—Fe2—C40—C44	150.0 (11)
C7—C8—C9—Fe1	59.2 (5)	C36—Fe2—C40—C44	−44.4 (8)
C5—Fe1—C9—C8	−161.7 (8)	C44—C40—C41—C42	1.5 (8)
C6—Fe1—C9—C8	−81.4 (5)	Fe2—C40—C41—C42	60.2 (5)
C4—Fe1—C9—C8	164.7 (5)	C44—C40—C41—Fe2	−58.7 (4)
C7—Fe1—C9—C8	−37.0 (5)	C43—Fe2—C41—C42	−37.8 (5)
C1—Fe1—C9—C8	47.2 (9)	C38—Fe2—C41—C42	110.8 (5)
C2—Fe1—C9—C8	82.1 (6)	C39—Fe2—C41—C42	156.8 (4)
C10—Fe1—C9—C8	−120.2 (6)	C44—Fe2—C41—C42	−81.1 (5)
C3—Fe1—C9—C8	125.5 (5)	C35—Fe2—C41—C42	−166.0 (6)
C5—Fe1—C9—C10	−41.5 (10)	C40—Fe2—C41—C42	−118.9 (6)
C6—Fe1—C9—C10	38.8 (3)	C37—Fe2—C41—C42	69.9 (5)
C4—Fe1—C9—C10	−75.0 (4)	C36—Fe2—C41—C42	34.7 (11)
C7—Fe1—C9—C10	83.2 (4)	C43—Fe2—C41—C40	81.1 (5)
C8—Fe1—C9—C10	120.2 (6)	C38—Fe2—C41—C40	−130.3 (4)
C1—Fe1—C9—C10	167.4 (6)	C42—Fe2—C41—C40	118.9 (6)
C2—Fe1—C9—C10	−157.7 (3)	C39—Fe2—C41—C40	−84.2 (5)
C3—Fe1—C9—C10	−114.3 (4)	C44—Fe2—C41—C40	37.8 (4)
C7—C6—C10—C9	1.0 (5)	C35—Fe2—C41—C40	−47.1 (8)
Fe1—C6—C10—C9	59.9 (3)	C37—Fe2—C41—C40	−171.2 (4)
C7—C6—C10—C11	−179.8 (5)	C36—Fe2—C41—C40	153.6 (8)
Fe1—C6—C10—C11	−121.0 (5)	C40—C41—C42—C43	−1.5 (8)
C7—C6—C10—Fe1	−58.9 (4)	Fe2—C41—C42—C43	59.3 (4)
C8—C9—C10—C6	−1.0 (6)	C40—C41—C42—Fe2	−60.8 (5)
Fe1—C9—C10—C6	−60.3 (3)	C43—Fe2—C42—C41	119.5 (6)
C8—C9—C10—C11	179.8 (5)	C38—Fe2—C42—C41	−87.8 (5)
Fe1—C9—C10—C11	120.6 (4)	C39—Fe2—C42—C41	−50.1 (8)
C8—C9—C10—Fe1	59.3 (4)	C44—Fe2—C42—C41	82.0 (5)
C5—Fe1—C10—C6	−77.3 (5)	C35—Fe2—C42—C41	156.7 (9)
C4—Fe1—C10—C6	−118.5 (4)	C40—Fe2—C42—C41	37.7 (4)
C7—Fe1—C10—C6	37.2 (5)	C37—Fe2—C42—C41	−129.0 (4)
C9—Fe1—C10—C6	116.3 (5)	C36—Fe2—C42—C41	−168.6 (4)
C8—Fe1—C10—C6	80.2 (5)	C38—Fe2—C42—C43	152.8 (5)
C1—Fe1—C10—C6	−46.7 (9)	C41—Fe2—C42—C43	−119.5 (6)
C2—Fe1—C10—C6	168.0 (6)	C39—Fe2—C42—C43	−169.6 (6)
C3—Fe1—C10—C6	−158.9 (4)	C44—Fe2—C42—C43	−37.5 (4)
C5—Fe1—C10—C9	166.4 (4)	C35—Fe2—C42—C43	37.2 (13)
C6—Fe1—C10—C9	−116.3 (5)	C40—Fe2—C42—C43	−81.7 (5)
C4—Fe1—C10—C9	125.2 (4)	C37—Fe2—C42—C43	111.6 (5)
C7—Fe1—C10—C9	−79.1 (5)	C36—Fe2—C42—C43	72.0 (5)
C8—Fe1—C10—C9	−36.1 (5)	C41—C42—C43—C44	0.8 (8)
C1—Fe1—C10—C9	−163.0 (8)	Fe2—C42—C43—C44	60.7 (5)
C2—Fe1—C10—C9	51.7 (7)	C41—C42—C43—Fe2	−59.8 (5)
C3—Fe1—C10—C9	84.8 (4)	C38—Fe2—C43—C44	−174.0 (5)
C5—Fe1—C10—C11	48.4 (5)	C42—Fe2—C43—C44	−118.7 (6)
C6—Fe1—C10—C11	125.7 (6)	C41—Fe2—C43—C44	−81.8 (5)
C4—Fe1—C10—C11	7.2 (5)	C39—Fe2—C43—C44	44.1 (13)
C7—Fe1—C10—C11	162.9 (6)	C35—Fe2—C43—C44	72.9 (5)
C9—Fe1—C10—C11	−118.0 (6)	C40—Fe2—C43—C44	−38.4 (4)
C8—Fe1—C10—C11	−154.1 (6)	C37—Fe2—C43—C44	155.9 (4)
C1—Fe1—C10—C11	79.0 (9)	C36—Fe2—C43—C44	114.4 (4)
C2—Fe1—C10—C11	−66.3 (8)	C38—Fe2—C43—C42	−55.3 (7)
C3—Fe1—C10—C11	−33.3 (6)	C41—Fe2—C43—C42	36.9 (4)
C12—N4—C11—C10	172.7 (4)	C39—Fe2—C43—C42	162.8 (10)
C20—N4—C11—C10	−58.8 (5)	C44—Fe2—C43—C42	118.7 (6)
C6—C10—C11—N4	−87.2 (6)	C35—Fe2—C43—C42	−168.4 (4)
C9—C10—C11—N4	91.7 (5)	C40—Fe2—C43—C42	80.3 (5)
Fe1—C10—C11—N4	179.7 (3)	C37—Fe2—C43—C42	−85.4 (5)
C11—N4—C12—C13	−73.5 (5)	C36—Fe2—C43—C42	−126.9 (5)
C20—N4—C12—C13	158.3 (4)	C42—C43—C44—C40	0.1 (7)
N4—C12—C13—C18	−66.0 (6)	Fe2—C43—C44—C40	60.2 (4)
N4—C12—C13—C14	113.2 (5)	C42—C43—C44—Fe2	−60.1 (5)
C18—C13—C14—C15	−2.7 (8)	C41—C40—C44—C43	−1.0 (7)
C12—C13—C14—C15	178.1 (5)	Fe2—C40—C44—C43	−59.6 (5)
C13—C14—C15—C16	2.6 (9)	C41—C40—C44—Fe2	58.6 (5)
C14—C15—C16—C17	−1.4 (10)	C38—Fe2—C44—C43	164.7 (13)
C15—C16—C17—C18	0.5 (9)	C42—Fe2—C44—C43	38.2 (4)
C15—C16—C17—C19	179.7 (6)	C41—Fe2—C44—C43	81.1 (5)
C16—C17—C18—C13	−0.7 (9)	C39—Fe2—C44—C43	−166.3 (4)
C19—C17—C18—C13	−179.9 (6)	C35—Fe2—C44—C43	−126.2 (4)
C14—C13—C18—C17	1.8 (8)	C40—Fe2—C44—C43	118.3 (6)
C12—C13—C18—C17	−179.0 (5)	C37—Fe2—C44—C43	−47.7 (7)
C11—N4—C20—C21	−64.2 (6)	C36—Fe2—C44—C43	−82.3 (5)
C12—N4—C20—C21	61.2 (5)	C43—Fe2—C44—C40	−118.3 (6)
C11—N4—C20—C25	169.6 (4)	C38—Fe2—C44—C40	46.4 (15)
C12—N4—C20—C25	−65.0 (5)	C42—Fe2—C44—C40	−80.2 (5)
N4—C20—C21—C22	170.3 (5)	C41—Fe2—C44—C40	−37.3 (4)
C25—C20—C21—C22	−61.1 (7)	C39—Fe2—C44—C40	75.4 (5)
C20—C21—C22—C23	59.6 (8)	C35—Fe2—C44—C40	115.5 (4)
C21—C22—C23—C24	−57.5 (9)	C37—Fe2—C44—C40	−166.1 (5)
C22—C23—C24—C25	57.8 (8)	C36—Fe2—C44—C40	159.4 (4)
C34—N3—C25—C20	162.1 (4)		

### Hydrogen-bond geometry (Å, °)

**Table:**

*D*—H···*A*	*D*—H	H···*A*	*D*···*A*	*D*—H···*A*
C21—H21A···N1^i^	0.97	2.59	3.486 (9)	154
C37—H37···N2^ii^	0.93	2.59	3.364 (11)	141

Symmetry codes: (i) *x*, *y*, *z*−1; (ii) −*x*, *y*−1/2, −*z*+2.
